# Supplementary material for: Optimal minimal residual disease threshold in pediatric acute myeloid leukemia: A retrospective cohort study based on the TARGET database
Source: PLoS Med. 2026 May 8;23(5):e1005088. doi: 10.1371/journal.pmed.1005088 (PMC13155632; doi:10.1371/journal.pmed.1005088)
Supplement: S1 Code — (ZIP) [file pmed.1005088.s002.zip › S2 code/PROJ8_2_tbl1/PROJ8_2_tbl1.htm]

## Ô¤²âÄ£ÐÍÓëROC·ÖÎö

Please use EmpowerStats ver. 2.2 or 3.0 since R packages updated, results from this version may be inaccurate.
Outcome:
First Event
Time:
X28

Model 0: Multiple Fractional Polynomial model from observed data
0.52478\*I((X25 + 0.1)^0.5)

|  |  |  |  |  |  |  |
| --- | --- | --- | --- | --- | --- | --- |
|  | coeff. | se. | HR | Low 95%CI | High 95%CI | P value |
| I((X25 + 0.1)^0.5) | 0.5248 | 0.0276 | 1.6901 | 1.6011 | 1.7841 | <0.0001 |

rsq = 0.1661, maxrsq = 0.9989
, Log likelihood -4012.2067

|  |  |  |  |  |  |  |
| --- | --- | --- | --- | --- | --- | --- |
| concordant | discordant | tied.risk | tied.time | std(c-d) | C | se(C) |
| 122160 | 28907 | 386069 | 1231 | 5773.4419 | 0.5868 | 0.0054 |

Model 1: Full model from observed data
0.06596\*X25

|  |  |  |  |  |  |  |
| --- | --- | --- | --- | --- | --- | --- |
|  | coeff. | se. | HR | Low 95%CI | High 95%CI | P value |
| X25 | 0.0660 | 0.0036 | 1.0682 | 1.0606 | 1.0758 | <0.0001 |

rsq = 0.1436, maxrsq = 0.9989
, Log likelihood -4028.2155

|  |  |  |  |  |  |  |
| --- | --- | --- | --- | --- | --- | --- |
| concordant | discordant | tied.risk | tied.time | std(c-d) | C | se(C) |
| 122160 | 28907 | 386069 | 1231 | 5773.4419 | 0.5868 | 0.0054 |

|  |  |  |  |  |  |  |  |  |  |
| --- | --- | --- | --- | --- | --- | --- | --- | --- | --- |
| Obs | Events | Model L.R. | d.f. | P.value | Score | Score P | R2 | g | gr |
| 1205 | 611 | 186.84 | 1 | <0.0001 | 809.6058 | 0 | 0.1438 | 0.2419 | 1.2736 |

Ô¤²âÄ£ÐÍROCÇúÏß·ÖÎö¼°×î¼ÑãÐÖµ·ÖÎö

|  |  |  |  |  |  |  |  |  |  |  |
| --- | --- | --- | --- | --- | --- | --- | --- | --- | --- | --- |
| Model | Time | Best.cut.X | Best.cut.sens | Best.cut.spec | Cases | N.survivor | N.censored | Cum.incidence | Surv.prob | AUC |
| mfp | 0.38 | 3.33808934203404 | 0.574 | 0.951 | 122 | 1077 | 6 | 0.101 | 0.897 | 0.775 |
| mfp | 0.56 | 4.14768252409385 | 0.445 | 0.935 | 182 | 1012 | 11 | 0.151 | 0.8428 | 0.698 |
| mfp | 0.7 | 4.14768252409385 | 0.359 | 0.939 | 242 | 952 | 11 | 0.201 | 0.7929 | 0.654 |
| mfp | 0.81 | 4.17826797305075 | 0.318 | 0.943 | 305 | 887 | 13 | 0.254 | 0.7395 | 0.634 |
| mfp | 0.92 | 4.17826797305075 | 0.278 | 0.945 | 363 | 831 | 11 | 0.302 | 0.6937 | 0.615 |
| mfp | 1.09 | 4.17826797305075 | 0.246 | 0.945 | 426 | 767 | 12 | 0.355 | 0.6435 | 0.599 |
| mfp | 1.38 | 4.17826797305075 | 0.223 | 0.947 | 487 | 694 | 24 | 0.406 | 0.5902 | 0.587 |
| Full | 0.38 | 3.1063075133837 | 0.574 | 0.951 | 122 | 1077 | 6 | 0.101 | 0.897 | 0.775 |
| Full | 0.56 | 4.6894124439072 | 0.445 | 0.935 | 182 | 1012 | 11 | 0.151 | 0.8428 | 0.698 |
| Full | 0.7 | 4.6894124439072 | 0.359 | 0.939 | 242 | 952 | 11 | 0.201 | 0.7929 | 0.654 |
| Full | 0.81 | 4.75537514934568 | 0.318 | 0.943 | 305 | 887 | 13 | 0.254 | 0.7395 | 0.634 |
| Full | 0.92 | 4.75537514934568 | 0.278 | 0.945 | 363 | 831 | 11 | 0.302 | 0.6937 | 0.615 |
| Full | 1.09 | 4.75537514934568 | 0.246 | 0.945 | 426 | 767 | 12 | 0.355 | 0.6435 | 0.599 |
| Full | 1.38 | 4.75537514934568 | 0.223 | 0.947 | 487 | 694 | 24 | 0.406 | 0.5902 | 0.587 |

×î¼ÑãÐÖµÈ¡Ãô¸Ð¶È+ÌØÒì¶È×î´óµÄ·Ö½çÖµ¡£¸÷·Ö½çµã¶ÔÓ¦µÄÃô¸Ð¶ÈÌØÒì±£´æÔÚROCÊä³öÎÄ¼þ£¨.xls£©Àï
Inverse Probability of Censoring Weighting (IPCW) estimates of Cumulative/Dynamic time-dependent ROC curve.
